# Focus on the proximal femur

**DOI:** 10.1007/s00068-024-02502-1

**Published:** 2024-03-28

**Authors:** Klemens Horst, Frank Hildebrand

**Affiliations:** Department of Orthopedics, Trauma and Reconstructive Surgery, University Hospital RWTH Aachen, Pauwelsstraße 30 52074, Aachen, Germany

Proximal femur fractures (PFF), often associated with osteoporosis and falls, represent a significant healthcare challenge, especially among the aging population. As the global demographic undergoes a shift towards an older age structure, the incidence of these fractures is on the rise [1]. However, recent advancements have brought about promising developments in the diagnosis, treatment and rehabilitation of PFF. This issue of the European Journal of Trauma and Emergency Surgery therefore focuses on recent aspects of PFF in the elderly.

Typically, PFF are managed surgically. Joint-preserving techniques, such as percutaneous fixation, extramedullary devices (i.e. dynamic hip screw or femoral neck fixation system) and intramedullary nailing, are widely used. These approaches minimize tissue disruption, reduce postoperative pain and accelerate the rehabilitation process. However, literature on the development of complications in geriatric populations treated with such minimally invasive techniques is sparse. Robioneck et al. therefore analysed a cohort of 202 patients with trochanteric fractures and found that osteoporosis was not significantly associated with implant failure while good fracture reduction and optimal implant positioning remain of the highest importance [2]. As dementia and frailty have been associated with worse outcomes in patients with hip fractures, Loannidis et al. investigated the predictive relationship between these co-morbidities and in-hospital mortality after hip fracture surgery [3]. The authors included 216,395 patients and found that dementia functions as a surrogate for frailty when predicting in-hospital mortality in hip fracture patients. Thus, they concluded that early frailty screening is important to improve care pathways but also to set realistic standards regarding expected outcomes.

Along with increasing life expectancy, patients suffer from multiple co-morbidities. A significant number of trauma patients are under treatment with anticoagulants for cardiovascular or cerebrovascular disease. Current literature reports that one third of all patients with PFF are under treatment with anticoagulative medication [4]. Against this background Kim et al. focuses on perioperative outcomes in patients that undergo urgent surgery due to a PFF [5]. The authors found that patients with platelet aggregation inhibitors could undergo surgery safely without delay, which led to no significant difference in operation time, postoperative complication risk, perioperative blood transfusion and variables related to bleeding risk. Thus, it was concluded that it is unnecessary to delay surgery for patients with PFF who are treated with platelet aggregation inhibitors. As no comprehensive guidelines for the perioperative management of patients under current treatment with direct oral anticoagulants (DOAC) are available, Marc Maegele summarizes the current evidence on the safe time window for surgery in patients in patients with PFF [6]. Furthermore, the author outlines the possible therapeutic options if emergency DOAC reversal becomes necessary. In summary, for most patients with PFF that are treated with DOAC, early surgery appears safe. Although there may be an increase in the need for blood products, data on this topic are not conclusive. Work-up on remaining anticoagulant activity and potential reversal should be restricted to patients at risk for bleeding complications. Maegele also concludes that preinjury DOAC use should not routinely delay surgery in patients with PFF.

In conclusion, the presented articles reflect some of the most important problems in patients with fractures to the proximal femur and highlights awareness of the development of peri- and postoperative complications in this vulnerable but increasing patient cohort. Moreover, a summary of current data regarding treatment of patients with PFF under anticoagulative therapy helps to reduce uncertainty when operative treatment is indicated.

We hope to have made a suitable selection of topics around the proximal femur that the reader will enjoy and that will help to improve patient outcomes.
